# Improved Quantification of Circulating Tumor DNA in Translocation‐Associated Myxoid Liposarcoma by Simultaneous Detection of Breakpoints and Single Nucleotide Variants

**DOI:** 10.1002/cam4.70704

**Published:** 2025-02-20

**Authors:** A. Schmid, U. Lausch, A. Runkel, J. Kiefer, T. Pauli, M. Boerries, B. Bogner, S. U. Eisenhardt, D. Braig

**Affiliations:** ^1^ Department of Plastic and Hand Surgery Medical Center ‐ University of Freiburg, Faculty of Medicine, University of Freiburg Freiburg Germany; ^2^ Institute of Medical Bioinformatics and Systems Medicine Medical Center–University of Freiburg, Faculty of Medicine, University of Freiburg Freiburg Germany; ^3^ Department of Radiology Medical Center–University of Freiburg, Faculty of Medicine, University of Freiburg Freiburg Germany

**Keywords:** diagnostic biomarker, liquid biopsy, myxoid liposarcoma, next‐generation sequencing, soft tissue sarcoma

## Abstract

**Background:**

Myxoid liposarcomas (MLS) can exhibit a disseminated metastatic pattern, necessitating extensive diagnostics during follow‐up. With no tumor markers available, early diagnosis of recurrences and tumor monitoring is difficult. The detection of circulating tumor DNA (ctDNA; liquid biopsy) in MLS with the characteristic translocations t(12;16) and t(12;22) can provide an additional diagnostic. However, due to the often very low tumor fraction, distinguishing actual tumor variants from sequencing artifacts remains a key challenge.

**Methods:**

Using MLS as a model for translocation‐driven tumors, this study evaluates a refined analytical approach for detecting both single nucleotide variants (SNVs) and structural variants (SVs) with the highest possible sensitivity and specificity. Different analysis pipelines using Unique Molecular Identifiers (UMIs) were compared in dilution series of tumor DNA from MLS patients (*n* = 11) and a cell line. The results were validated on plasma samples (*n* = 36) from two MLS patients and one patient with Synovial Sarcoma (SS).

**Results:**

In dilution series, the use of UMIs significantly reduced false positive events in SNV analysis while maintaining high sensitivity without significant differences. In SV analysis, the effect of UMIs was not consistently detectable, as some dilution series exhibited no false positive events even without UMI correction. Additional filter criteria further improved specificity without significantly compromising assay sensitivity. Validation on patient plasma samples confirmed these findings, demonstrating the advantages of the differentiated analytical approach.

**Conclusion:**

By integrating a refined analytical approach for SNVs and SVs, we achieved reliable ctDNA detection that corresponded with the clinical course of the patients’ disease. This method enables non‐invasive tumor detection in translocation‐driven tumors with low mutational burden and can easily be adapted into routine clinical diagnostics.

## Graphical abstract

Due to a disseminated metastatic pattern and no established tumor markers, early detection and monitoring of recurrences of myxoid liposarcomas are challenging. Liquid biopsy with the detection of circulating tumor DNA (ctDNA) can support diagnostics. Especially low concentrations of ctDNA can make the detection of small tumors difficult. This approach significantly improves the sensitivity and specificity of MLS liquid biopsy using unique molecular identifiers and a sophisticated bioinformatic analysis.

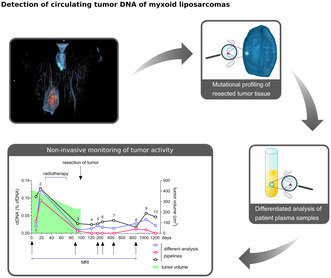

**Abbreviations**
bp: base pairs
CCCF: Comprehensive Cancer Center Freiburg
cfDNA: cell‐free DNA
CT: Computer Tomography
ctDNA: circulating tumor DNA
FFPE: Formalin‐Fixed Paraffin‐Embedded tissue
MLS: myxoid liposarcoma
MRD: minimal residual disease
MRI: Magnetic Resonance Imaging
NGS: next‐generation sequencing
nt: nucleotide
PCR: polymerase chain reaction
SNVs: single nucleotide variants
SOPs: standard operating procedures
SS: synovial sarcoma
STS: soft tissue sarcoma
SVs: structural variants
UMI: unique molecular identifier
VAF: variant allele frequency
WES: Whole‐Exome Sequencing

## 1 Introduction

High‐grade soft tissue sarcomas (STS) account for less than 1% of human malignancies and are associated with a high treatment‐related morbidity and a high mortality rate. About 50% of patients with high‐grade STS experience relapse after multimodal treatment of a localized primary tumor, and many will eventually succumb to their disease [1, 2].

Follow‐up examinations and imaging are performed at close intervals as timely detection and treatment of local recurrence and distant metastases enhance survival [3]. According to current guidelines, imaging includes magnetic resonance imaging (MRI) of the primary tumor region and computed tomography (CT) of the lungs [4]. As metastases to extrapulmonary sites can be observed in up to 30% of recurrences, some advocate for more extensive imaging in selected subtypes, for example, whole‐body MRI or a combination of various imaging modalities in Myxoid Liposarcoma (MLS) [5]. Establishing biomarkers in STS is difficult due to their rarity and diverse genetic nature [6]. Currently, there are no established biomarkers that aid in cancer surveillance and help us monitor treatment response.

Noninvasive diagnostic by genotyping of circulating cell‐free DNA (cfDNA) might improve tumor detection in sarcomas independent of their anatomic localization. It utilizes only a small blood specimen taken from the peripheral circulation and quantifies tumor‐derived DNA fragments (circulating tumor DNA, ctDNA) therein [6]. The concentration of ctDNA correlates with tumor volume as well as tumor stage and can be used to track tumor dynamics and predict minimal residual disease (MRD). Quantification of ctDNA in the peripheral circulation might, thus, support or even surpass common imaging modalities in their sensitivity and specificity to detect tumors [7].

Translocation‐associated sarcomas account for about one fourth of STS. Subtype‐specific reciprocal translocations lead to the initiation and proliferation of tumor cells. Besides, these tumors harbor only a few additional hotspot mutations, which makes it difficult to identify sufficient target mutations for ctDNA quantification. MLS is one of the most common STS that is characterized by specific genetic translocations t (12;16) (q13;p16) and t (12;22)(q13;22q11‐12) which lead to the fusion of the DDIT3 gene to FUS or EWS [8, 9]. Despite these specific translocations, only a few additional hotspot mutations have been identified in MLS. Promoter mutations in TERT have been described in up to 80% of MLS and seem to occur secondarily to the initiating translocation events [10, 11, 12].

Activation of the PI3K/Akt pathway is another recurrent genetic event, and alterations in PIK3CA, Akt, and PTEN could be identified in 27% of cases [13, 14].

Beyond these, even whole‐exome sequencing (WES) has not yielded additional recurrently altered genes [15]. Thus, standard tumor panels for NGS are not applicable for mutation profiling in MLS [16].

We have established targeted next‐generation sequencing (NGS) based assays for MLS that can identify breakpoints and recurrent hotspot mutations with a variant allele frequency of < 0.05%. Adding additional mutations obtained by WES enhanced the sensitivity of the assay. ctDNA could be detected in the peripheral blood, correlated with disease activity, and identified MRD and distant recurrence in selected patients [17, 18]. Implementing this assay in routine diagnostics requires stringent standard operating procedures (SOPs) including a robust bioinformatic analysis. This pipeline needs to extract single nucleotide variants (SNVs) and breakpoints (structural variants, SVs) at base‐pair resolution during tumor mutational profiling and needs to quantify ctDNA harboring these mutations at the highest sensitivity and specificity possible. We evaluated different bioinformatic pipelines for the quantification of ctDNA in translocation‐associated MLS in several dilution series as well as patients plasma samples to set the foundation for routine noninvasive tumor monitoring of translocation‐associated sarcomas.

## 2 Materials and Methods

### 2.1 Study Population

Samples in this study were obtained from 11 MLS patients and one patient with synovial sarcoma (SS). All of them were treated at the Comprehensive Cancer Center Freiburg (CCCF) (Freiburg, Germany). An additional cell line was used (MLS402; RRID:CVCL_S813) [12]. For mutational profiling and dilution series, formalin‐fixed paraffin‐embedded (FFPE) and fresh frozen tissue, as well as whole blood, was available. Corresponding plasma samples of these patients and one healthy control sample were used to quantify cfDNA.

### 2.2 Ethics, Consent and Permission

The Ethics Committee of the Albert‐Ludwigs‐University of Freiburg, Germany, approved the study (study number: 236/16). The design and performance of the study are in accordance with the Declaration of Helsinki. Participants signed informed consent before inclusion, allowing analysis of tumor tissue, blood samples, and clinical data.

### 2.3 Blood and Tissue Sampling

Collection of blood samples was performed by puncture of an uncongested vein in the cubital fossa using a 21G butterfly needle. The first 3 mL was discarded. Nine milliliters was withdrawn into K_3_EDTA tubes (Sarstedt AG & Co., Nümbrecht, Germany) and processed within the next 2 h. Therefore, blood samples were double centrifuged for 15 min at 2,500 g at 22°C. Plasma aliquots of 2 mL were then stored in FluidX cryotubes (Brooks Life Sciences, Chelmsford, MA, USA) at −80°C until further use.

### 2.4 Isolation of DNA From Fresh Frozen and FFPE Tissue

Isolation of DNA from fresh frozen tissue was performed using the DNeasy Blood & Tissue Kit (Qiagen GmbH, Hilden, Germany) according to the manufacturer's instructions in the *Purification of Total DNA from Animal Tissue* (*Spin‐Column Protocol*) protocol. DNA in FFPE tissue was isolated using the QIAamp DNA FFPE Tissue Kit (Qiagen GmbH, Hilden, Germany) according to the instructions of the *QIAamp DNA FFPE Tissue Handbook*. Eight sections, each 10 μm (approximately 25 mg), were digested with Proteinase K at 56°C for 72 h before continuing the protocol. Elution was performed with 80‐μL nuclease‐free water.

### 2.5 Isolation of DNA From Whole Blood and Leukocytes

DNA from whole blood and leukocytes was isolated following the protocol *Purification of total DNA from animal blood or cells* (*spin‐column protocol*) using the DNeasy Blood & Tissue Kit (Qiagen GmbH, Hilden, Germany). As starting material, 100 μL of whole blood was used. All the following steps were performed according to the manual. The isolated DNA was then eluted in 200‐μL nuclease‐free water. Final DNA concentration was measured using the Invitrogen Qubit 3 Fluorometer.

### 2.6 Isolation of Cell‐Free DNA (cfDNA)

The QIAamp Circulating Nucleic Acid Kit (Qiagen GmbH, Hilden, Germany) was used for the extraction of cell‐free DNA from plasma samples. As starting material, approx. 4.5 mL of plasma was thawed and the volume was topped up to 5 mL with PBS. Further extraction was carried out according to the manufacturer's instructions from the protocol “Purification of Circulating Nucleic Acids from 4 mL or 5 mL Serum or Plasma” by using the QIAvac 24 Plus System (Qiagen GmbH, Hilden, Germany). For better control of the vacuum acting on the samples during extraction, so‐called VacValves were used. Elution of the isolated cfDNA was done with 30‐μL AVE buffer, followed by a repetition of the last step by adding the eluate a second time to the QIAamp Mini column, centrifugation, and elution with 30‐μL AVE buffer for higher yield.

### 2.7 Library Preparation of Plasma Samples Using Unique Molecular Identifiers

Libraries of plasma samples and fragmented matched normal samples were prepared with adapters containing UMIs. For this purpose, the SMARTer ThruPLEX Tag‐seq Kit (Takara Bio USA Inc., Mountain View, CA, USA) was used. In this preparation, adaptors containing six random oligonucleotides, so‐called unique molecular identifiers(UMI), were ligated to the 5′ and 3′ ends of each DNA fragment, separated by a variable stem sequence (8–11 nt stem). A sample‐specific index (8 nt Index) allowed multiplexing of samples. Preparation was performed according to the manufacturer's instructions following the SMARTer ThruPLEX Tag‐seq Library Preparation Protocol. As starting material 10 ng of the previously extracted cfDNA or fragmented matched normal DNA was used. Purification was done using Agencourt AMPure XP—PCR Purification beads (Beckman‐Coulter, Brea, CA, USA), again following the manufacturer's instructions. In the final step, the libraries were then eluted in 33 μL 0.1 X TE buffer.

### 2.8 Target Enrichment for cfDNA Libraries and Dilution Series

For target enrichment, we have designed and published an MLS‐specific enrichment panel (*standard panel*) using xGen Lockdown Probes (Integrated DNA Technologies, Coralville, IA, USA) covering 36,541 base pairs (bp). This includes breakpoint regions and exons of genes that have been described in the literature as hotspot areas in MLS, with a reported mutation frequency of at least 5%.

Based on mutations that were identified in WES of two patients, additional patient‐specific enrichment panels were created (so‐called *combined panels*), in which the *standard panel* was supplemented by the respective mutations [18].

Target enrichment and hybridization captures were then performed with the xGen Hybridization and Wash Kit (IDT, Coralville, IA, USA) following the manufacturer's instructions of the *xGen hybridization capture of DNA libraries for NGS target enrichment—Tube protocol*. Samples were merged into pools with equal amounts between 100 and 250 ng per library. First, a hybridization capture was performed with incubation at 65°C for 4 h, followed by PCR with 16 cycles. For higher on‐target rates, a second hybridization capture was performed with incubation at 65°C for 4 h and postcapture PCR with nine cycles. As starting material 500 ng of purified postcapture PCR fragments of the first capture was used [19]. Samples were purified using Agencourt AMPure XP beads (Beckman Coulter, Brea, CA, USA) and fragment length was then measured with Tape Station Agilent D500 (Agilent, Santa Clara, CA, USA). The exact DNA concentration of the cleaned‐up captured libraries was finally determined with qPCR LightCycler 480 System (Roche, Basel, Switzerland) following the manufacturer's instructions of the NEBNext Library Quant Kit for Illumina (New England Biolabs, Ipswich, MA, USA).

Samples were then sequenced with the Miseq system using Miseq V2 300‐cycle, paired‐end reads (Illumina Inc., San Diego, CA, USA).

### 2.9 Quantification of Cell‐Free DNA and Fragment Length

Quantity of DNA concentration was determined after relevant steps in library preparation, hybridization capture, and sequencing via an Invitrogen Qubit 3 Fluorometer (Invitrogen, Carlsbad, CA, USA) according to dsDNA HS Assay Kit *0.2–100 ng* protocol. Fragment length was assessed using the Fragment Analyzer system (Agilent, Santa Clara, CA, USA).

### 2.10 Bioinformatical Pipelines and Analysis

For UMI‐tagged libraries prepared with the SMARTer ThruPLEX Tag‐seq Kit, the *Curio Genomics* web platform was specifically recommended by the manufacturer as a bioinformatical tool for downstream sequencing analysis. First, the sequence reads from cfDNA samples were uploaded to the *Curio Genomics* web platform (www.curiogenomics.com). Alignment was performed using Bowtie2 (Galaxy version 2.5.3) [20] to the human genome (hg38). UMI family consensus reads were called, and SNVs were discovered using the *Curio Genomics* integrated analysis pipeline. Only families with at least three reads per family were considered. The consensus threshold was set to 60%.

**FIGURE 1:**
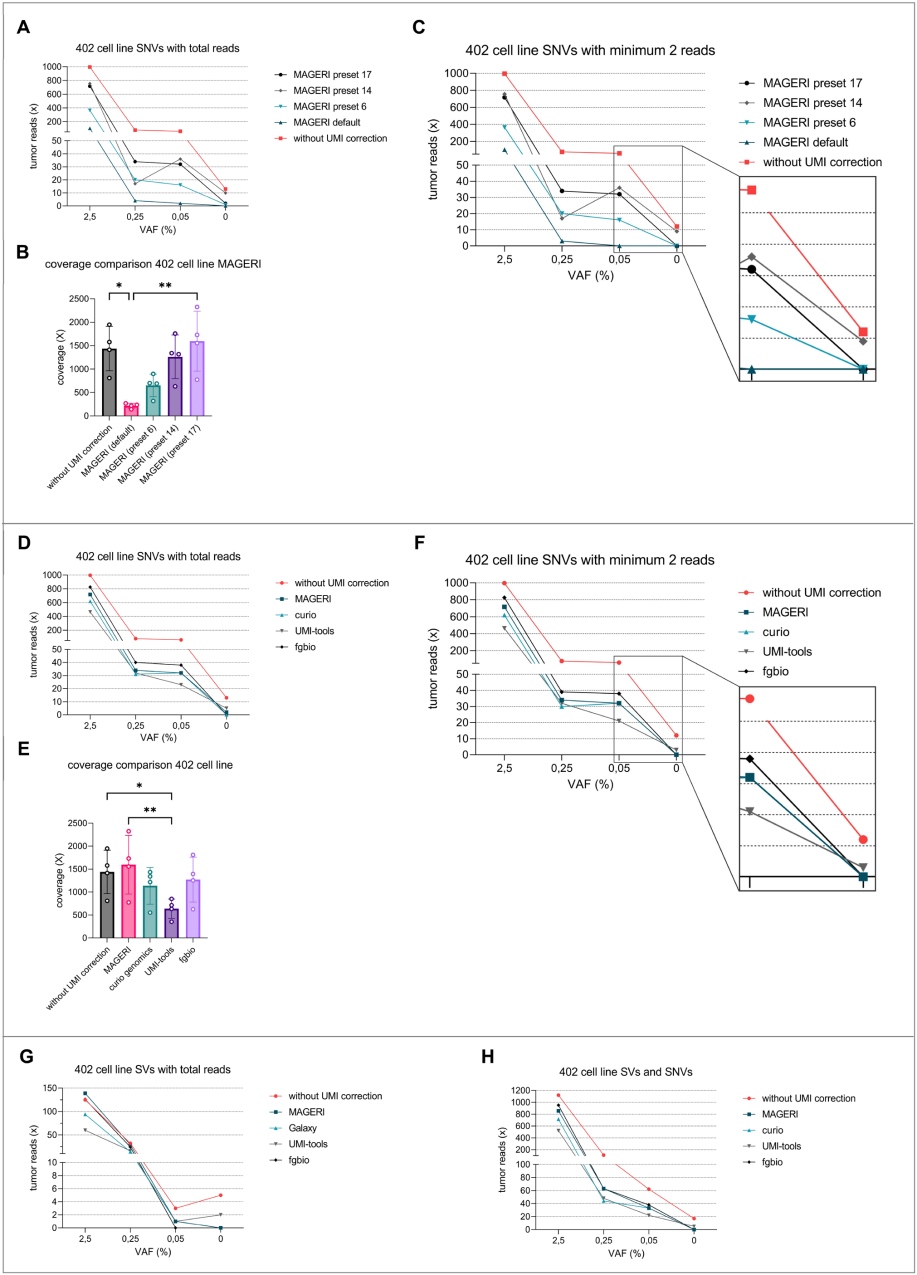
Comparison of bioinformatical pipelines analyzing cell‐line dilution series. Ten‐nanogram cfDNA of a single healthy donor was spiked with fragmented MLS 402 cell‐line DNA each to obtain dilution series with variant allele frequencies (VAF) of 2.5%, 0.25%, 0.05%, and 0%. Target enrichment was performed with an entity‐specific target enrichment panel (*MLS Standard Panel*). Paired‐end sequencing was performed to obtain information from the 5′ and 3′ ends of each fragment separately. (A) Samples were analyzed for single nucleotide variants (SNVs) with the *MAGERI* computational pipeline in different settings compared to analysis without UMI error correction. Results were then manually assessed with *IGV*, and each variant read was counted. The use of UMIs led to lower coverages but also reduced the ratio of false‐positive events in the control sample (VAF 0%). Sensitivity could be improved by changing the preset parameters. (B) Mean coverages after deduplication and consensus calling are shown. A significant decrease in coverage was observed in samples analyzed with *MAGERI* in the default preset (Friedman test). (C) Specificity was further improved by only considering variations with at least two variant reads. The total coverage of variant reads was not significantly affected. In terms of sensitivity and false positives, preset 17 showed the most promising results. (D—F) The same dilution series was then further analyzed for SNVs using different pipelines in different settings and again compared to the analysis without UMI error correction. The *fgbio* pipeline with single‐stranded consensus calling provided improved results in terms of sensitivity and false‐positive events. (E) Mean coverages after deduplication and consensus calling are shown. A significant decrease in coverage was observed in samples analyzed by *UMI tools* (Friedman test). (G) Additionally, the different pipelines were compared with respect to their performance in the detection of structural variants (SVs). For this analysis, each variant read was counted. Only the *fgbio* and *MAGERI* pipelines showed no false‐positive events. (H) This figure shows the combination of detected SVs (every variant read counted) and SNVs (minimum of two variant reads for counting). With this setup, few pipelines were able to detect low VAFs and still show no false‐positive reads.

*MAGERI* pipeline was used to analyze both SNVs and SVs. This tool provides a UMI‐analysis pipeline from UMI extraction up to consensus assembly and alignment (version 1.1.1) [21]. Various modifications of the default preset were tested on dilution series. The “positional” mode (M3) was selected for UMI extraction. The following parameters were changed in the preset file as an example for preset 17 (Figure 1) that was used for analyzing plasma samples: As *PreprocessorParameters* we used (1) an *umiQualThreshold* of 5, (2) the *minUmiMismatchRatio* was set to 66.6, and (3) *defaultOverseq* was set to 2 to increase the final coverage. Further modifications were made for Consensus assembly in *AssemblerParameters*: (4) the *offsetRange* was set to 3, the parameters for (5) *anchorRegio* and (6) *maxMMs* were changed to 6 each, (7) the *maxDroppedReadsRatio* was lowered to 0.2, and (8) *readLength* was set to 150 corresponding to the sequencing platform (Supporting Information preset_17_0.xml). The specific parameters used for each preset configuration can be provided upon reasonable request. For alignment, patient‐specific fasta files were constructed for each patient (Supporting Information). Only the regions that were covered by the respective patient‐specific *combined panels* were considered. In each case, a defined sequence upstream and downstream of the mutation was included in the reference file. Consensus reads were then aligned to these reference fasta files and afterward converted and sorted into BAM files using *SAMtools* (version 1.16.1) [22]. Analysis of SVs was performed the same way, except alignment was done to individually designed fasta files. These files contained patient‐specific translocation sequences, each covering 180 bp upstream and downstream of both breakpoints.

The analysis of SNVs and SVs was also performed using the *fgbio* pipeline (version 2.1.0) [23], where SNVs were again aligned using bwa mem (version 0.7.17‐r1188) [24] against the human genome (hg38) and SVs against the above‐described patient‐specific translocation sequences. The workflow was designed based on the *KAPA Universal UMI Adaptor* protocol (Roche, Basel, Switzerland). Consensus reads were generated as single‐stranded consensus with *CallMolecularConsensusReads*. Modifications of default settings were made again with a minimum three reads for consensus calling. The consensus reads were finally converted and sorted into BAM files using *SAMtools*.

For analysis of SNVs using *UMI‐tools*, samples were first uploaded to the *Galaxy web platform*. A workflow was established based on *UMI‐tools* recommendations (Galaxy version 1.1.2) [25]. UMIs were extracted according to adaptor specifications. Samples were then aligned to the human genome (hg38) with *bwa mem* and further processed using *SAMtools* by removing duplicate reads and sorting. Finally, reads were grouped with the identification of PCR duplicates by identifying clusters based on hamming distance and resolving networks by using the node counts.

As a control, the same samples were also analyzed without taking the UMIs into account. To do this, the UMIs were first separated together with the adaptor sequence using *je‐clip* (version 1.2) [26]. Again, SNVs were analyzed by alignment to the human genome (hg38) and SVs by alignment to the patient‐specific translocation sequences using *bwa mem*. The samples were then further processed using *SAMtools* by removing duplicate reads and sorting.

To verify the respective SVs, the reads processed according to the abovementioned schemes were evaluated again manually using IGV. Only reads in which the breakpoint sequence was mapped against the patient‐specific translocation sequence over a span of 15 bp upstream and downstream without artifacts were counted as variant reads. Two approaches were examined regarding the analysis of SNVs. In the first approach, every variant read was included, whereas in the second approach, only mutations with at least two variant reads were considered.

## 3 Results

### 3.1 ctDNA Quantification With MLS‐Specific Panel

The samples used for this study were generated and published in a previous publication [18]. We have designed a 36,541 bp MLS‐specific enrichment panel (*standard panel*) that covers the *TERT* promoter region, mutation hotspots in exons from seven genes with a reported mutation frequency of at least 5%, and introns of DDIT3, FUS, and EWS, where the t(12;16) and t(12;22) translocations occur. Mutational profiling of matched normal tumor tissue detected an average of 2.8 mutations per MLS tumor, which can subsequently be quantified in cfDNA [18].

To establish a robust bioinformatics pipeline for the analysis of patient plasma samples with equally high sensitivity and specificity, different analytical pipelines were tested. Therefore, we generated MLS cell‐line dilution series in cfDNA with exactly defined variant allele frequencies (VAF). Ten‐nanogram cfDNA of a single healthy donor was spiked with fragmented MLS 402 cell‐line DNA at VAFs of 2.5%, 0.25%, 0.05%, and 0%. Libraries were prepared as mentioned in methods, and target enrichment was performed with the MLS‐specific s*tandard panel*. Samples were analyzed for SNVs with the *MAGERI* computational pipeline in different preset settings compared to analysis without UMI error correction (Figure 1A–C).

Without UMI correction, positive reads with SNVs were detected in every sample ranging from 996 reads at 2.5% VAF to 13 reads at 0% VAF (control sample, Figure 1A). The average coverage without UMI correction was 1437 (Figure 1B). The analysis with *MAGERI* in its default parameter settings [21] showed considerably fewer positive reads with 102 reads at 2.5% VAF, 2 reads at 0.05% VAF, and 0 reads at 0% VAF, and thus a good reduction of false‐positive events in the control sample by the application of UMIs (Figure 1A). The average coverage was reduced to 216.2 and differed significantly compared to different preset settings (Figure 1B, Friedman test). The *MAGERI* pipeline in preset 17 showed the best results in terms of sensitivity with 32 reads at 0.05% VAF, 2 reads at 0% VAF (Figure 1A) and an average coverage of 1596 (Figure 1B).

To further improve specificity by reducing false‐positive events, each preset was analyzed using SNVs with at least two variant reads. At 0% VAF (control sample), 12 variant reads were still detected without UMI correction, while *MAGERI* showed no false‐positive reads in the default preset, preset 6, and preset 17 (Figure 1C). In terms of sensitivity and false‐positive events, preset 17 with at least two variant reads showed the most promising results (Figure 1C).

The same dilution series was then further analyzed for SNVs with different pipelines allowing for UMI error correction (*Curio Genomics, fgbio, UMI tools*). The results were compared to *MAGERI preset 17* and to the analysis without UMI correction (Figure 1D–F). Detection of SNVs at 0.05% VAF ranged between 23 reads with *UMI tools* and 38 reads with *fgbio*. At 0% VAF, *UMI tools* showed five positive reads, while both *fgbio* and *Curio Genomics* were negative for SNVs (Figure 1D). The mean coverage differed significantly, with *UMI tools* resulting in the lowest coverage of 683 (Figure 1E, Friedman test). Considering variants with at least two variant reads again reduced the ratio of false positives, with UMI tools showing three positive events at 0% VAF (Figure 1F). The *fgbio* pipeline with single‐stranded consensus calling delivered improved results in terms of sensitivity and false‐positive events compared to the three competitors.

Additionally, the different pipelines were compared in terms of performance in detecting the MLS‐specific translocations t(12;16) (Figure 1G). As the sequences of breakpoints are highly specific for each tumor, every variant read was counted. As *Curio Genomics* did not allow analysis of the breakpoints, a custom pipeline (*Galaxy*) was employed instead. *Galaxy*, *fgbio*, and *MAGERI* showed no false‐positive events at 0% VAF (control sample), whereas *UMI tools* and the analysis without UMI correction returned 2 and 5 positive reads, respectively. At 0.05% VAF, *fgbio* could not detect any tumor reads, while the detection with other pipelines ranged between 1 and 3 positive reads (without UMI correction) (Figure 1G).

Combining the analysis of SNVs and SVs, every pipeline was able to detect variant reads at 0.05% VAF (Figure 1H). Enabling UMI correction at 0.05% VAF, the analysis with fgbio resulted in the most detected reads (*n* = 38). In terms of false‐positive events, only *Curio Genomics*, *MAGERI*, and *fgbio* showed no false‐positive reads, and with that, the highest specificity.

### 3.2 Objectify False‐Positive Ratio With Additional Dilution Series

**FIGURE 2:**
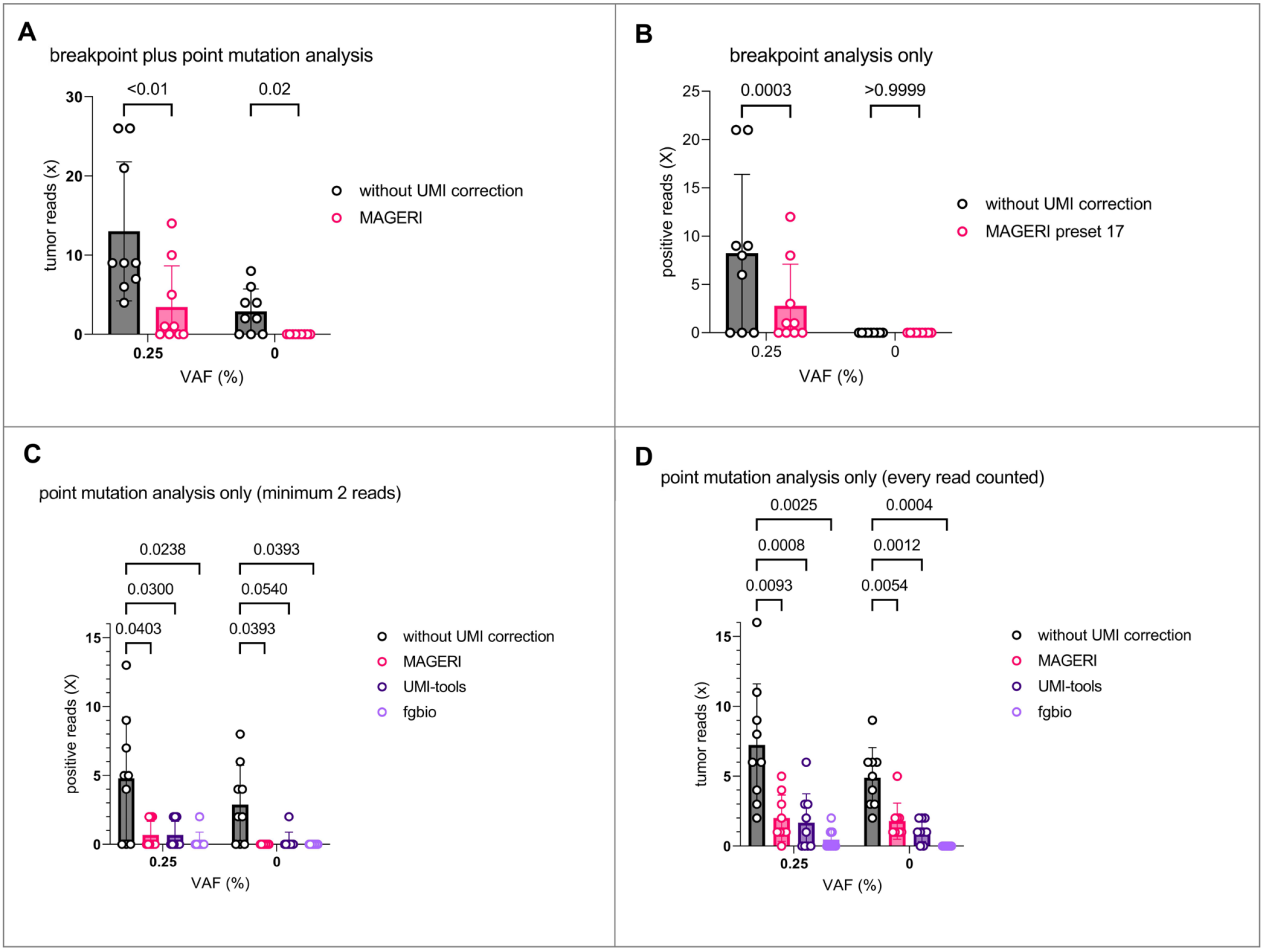
Objectify false‐positive ratio with additional dilution series. To further objectify the reduction in false‐positive reads, dilution series were compared with different analytical approaches. Tumor DNA from nine MLS patients was spiked in healthy control DNA to VAFs of 0.25% and 0%. (A) This figure shows the combined analysis of SVs and SNVs. For SVs, each variant read was counted, whereas for SNVs, only mutations with at least 2 variant reads were considered. The use of UMIs resulted in a significant reduction of positive tumor reads regardless of VAF. In the samples with a calculated VAF of 0%, the rate of false‐positive reads was reduced to zero. (B) Shows the same data set, but with breakpoint analysis only. Note that no false‐positive reads were detected either with or without UMI analysis. In contrast, the use of UMIs again led to a significant reduction of positive reads. (C‐D) Again, the data set from (A) is shown, but only point mutations are analyzed. Figure (C) includes only mutations for which at least 2 variant reads were detected (as described in A). For comparison, figure (D) shows the same analysis, but with every variant read included. The comparison clearly illustrates the effectiveness of this measure in analyzing the false‐positive rate.

To objectify the reduction of false‐positive reads again, various dilution series were compared with different analytical approaches. Target regions were enriched using the abovementioned MLS‐specific enrichment panel (*standard panel*). Tumor DNA of nine MLS patients was spiked with healthy control DNA to VAFs of 0.25 and 0% each. Initially, the samples were either analyzed without taking the UMIs into account or using *MAGERI* in preset 17 according to the method described above. When analyzing SVs, every variant read was included in the analysis, whereas when analyzing SNVs in both pipelines, only mutations with at least 2 variant reads were considered. At 0.25% VAF, an average of 13 reads was detected without UMI correction, while 3.4 reads were detected with *MAGERI* in preset 17. At 0% VAF, the analysis with MAGERI returned no false‐positive reads, whereas the analysis without UMI correction showed an average of 2.9 positive reads (Figure 2A). The use of UMIs led to a significant reduction in positive tumor reads regardless of the VAF (two‐way ANOVA). Most importantly, no false‐positive events were observed when enabling UMI correction (Figure 2A).

The effect of the UMI analysis was further differentiated between the analysis of SVs and the analysis of SNVs. The abovementioned dataset was, therefore, analyzed for SVs only (Figure 2B). Again, every variant read was considered. At 0.25% VAF, the analysis without UMI correction returned an average of 8.2 reads and the analysis with *MAGERI* showed 2.8 positive reads. At 0% VAF (control samples), both *MAGERI* and the analysis without UMI correction showed no false‐positive events (Figure 2B). The use of UMIs led to a significant reduction of positive tumor reads (two‐way ANOVA). In contrast, the use of UMIs had no significant influence on the ratio of observed false‐positive events.

Subsequently, the abovementioned dataset was again analyzed for SNVs only, this time with several different analysis pipelines. First, the samples were analyzed as described in the methods section by considering only mutations with at least 2 variant reads (Figure 2C). The use of UMIs again led to a significant reduction of positive tumor reads. Every analysis pipeline showed positive tumor reads in samples with a VAF of 0.25%, ranging from 1 positive sample with *fgbio* to 6 positive samples without UMI correction (Figure 2C). In contrast, at 0% VAF (control samples), only *MAGERI* and *fgbio* showed no false‐positive events, whereas the analysis without UMI correction returned 6 and the analysis with *UMI tools* returned 1 positive sample.

To demonstrate the influence of possible artifacts, the same data set was analyzed considering each variant read (Figure 2D). A reduction in positive reads using UMIs was confirmed here, too. However, significantly more false‐positive reads were detected with this method. Only the analysis with *fgbio* continued to show no false‐positive events. The comparison clearly illustrates the effectiveness of this measure in the analysis of the rate of false‐positive results (Figure 2D, two‐way ANOVA, Wilcoxon test).

### 3.3 Adding Mutations Obtained by Whole‐Exome Sequencing Increases Sensitivity

Tumor tissue and matched normal DNA from two MLS patients (patients 1 and 2) were subjected to WES. Tumor‐specific mutations were added to the mutations obtained from the *standard panel* to design a patient‐specific *combined panel*. Decreasing amounts of fragmented tumor DNA from these two MLS patients were spiked in 10 ng of fragmented matched normal DNA. This resulted in a dilution series with VAFs of 0.5%, 0.25%, 0.05%, and 0%. Libraries were enriched with patient‐specific *combined panels*, sequenced, and analyzed using the described pipelines.

**FIGURE 3:**
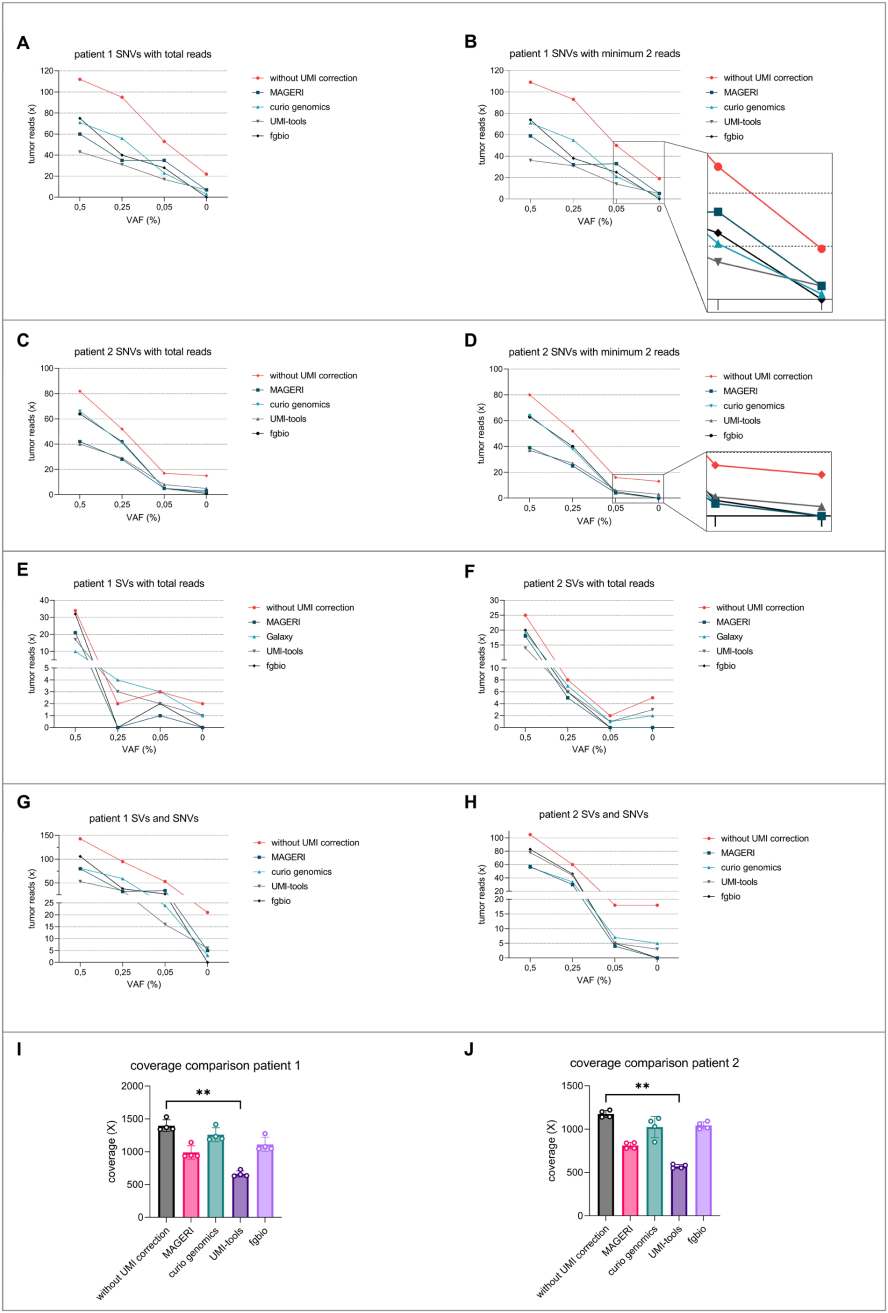
Dilution series after enrichment with additional mutations after whole‐exome sequencing. Decreasing amounts of tumor DNA from two MLS patients were spiked into 10‐ng cfDNA each from a healthy donor. This resulted in a dilution series with VAFs of 0.5%, 0.25%, 0.05%, and 0%. The samples were then pooled, sequenced, and analyzed using different pipelines and settings. (A, B) Tumor DNA from a patient with MLS recurrence was spiked into healthy cfDNA. Mutations were identified by WES, and target enrichment was performed using a patient‐specific target enrichment panel covering 26 different genomic regions. Shown are selections from different analytical pipelines analyzing SNVs with the inclusion of each variant read versus variants with at least two variant reads. (C, D) Tumor DNA from a patient with a primary femoral MLS and neoadjuvant radiotherapy was spiked into healthy cfDNA, resulting in a dilution series with VAFs of 0.5%, 0.25%, 0.05%, and 0%. Target enrichment was performed with a patient‐specific target enrichment panel after WES covering 19 different genomic regions. SNV analysis using UMI consensus calling again resulted in fewer false positives. Mutations were detected at a minimum VAF of 0.5%. (E, F) The samples from the previously described dilution series were additionally analyzed for SVs to compare different analysis pipelines. Again, each variant read was counted. Only two pipelines, *fgbio* and *MAGERI*, showed no false positives. (G, H) This figure shows the combination of detected SVs (every variant read counted) and SNVs (at least two variant reads). Only the analysis with the *fgbio* pipeline showed no false‐positive events, but it was still possible to detect variants with a VAF of 0.05%. (I, J) Mean coverages after deduplication and consensus calling are shown. A significant decrease in coverage was observed in samples analyzed with *UMI tools* (Friedman test).

Tumor DNA of patient 1 with local recurrences of his leg was enriched with a *combined panel* covering 26 different genomic regions. Depicted are different analytical pipelines analyzing SNVs with inclusion of every variant read (Figure 3A) in comparison to variants with at least two variant reads (Figure 3B). Only considering at least two variant reads substantially reduced the detected number of positive reads. Using this method without UMI correction, 19 positive reads were detected at 0% VAF instead of 22 positive reads. Comparing each pipeline at 0% VAF, only *fgbio* returned no false‐positive reads.

Next, tumor DNA of patient 2 with a primary MLS in the thigh after neoadjuvant radiation therapy was analyzed. Target enrichment was performed with a patient‐specific *combined panel* covering 19 genomic regions. Analysis of SNVs with UMI consensus calling with *fgbio* resulted in least false‐positive events (Figure 3C). Positive reads were detected at a minimal VAF of 0.05% in both patients (Figure 3A–D).

Both dilution series were additionally analyzed for SVs (t(12;16) breakpoint sequences, *n* = 2 per patient) comparing the described analysis pipelines (Figure 3E,F). Again, every breakpoint read was counted. Only two pipelines, *fgbio* and *MAGERI*, showed no false‐positive events at 0% VAF. Interestingly, no breakpoints were detectable at 0.25% VAF in patient 1 and 0.05% VAF in patient 2 in both the analyses with *MAGERI* and *fgbio* (Figure 3E,F). The combined analysis of SVs and SNVs further increased the number of tumor reads in each sample. Only the analysis with the *fgbio* pipeline showed no false‐positive events, yet the detection of variants at 0.05% VAF was consequently possible (Figure 3G,H). Mean coverages after deduplication and consensus calling were recorded for both dilution series. A significant decrease in coverage was detected with *UMI tools*, resulting in the lowest values (Figure 3I,J, Friedman test).

### 3.4 Performance of Different Pipelines Analyzing Patient Plasma Samples

The workflows and analysis pipelines were used to quantify SNVs and SVs in plasma samples of the two abovementioned patients (patients 1 and 2). Every sample was analyzed with *MAGERI*, *fgbio*, and without UMI correction. In the analysis of SVs, every read was considered, whereas for SNVs, only mutations with at least 2 variant reads were included.

**FIGURE 4:**
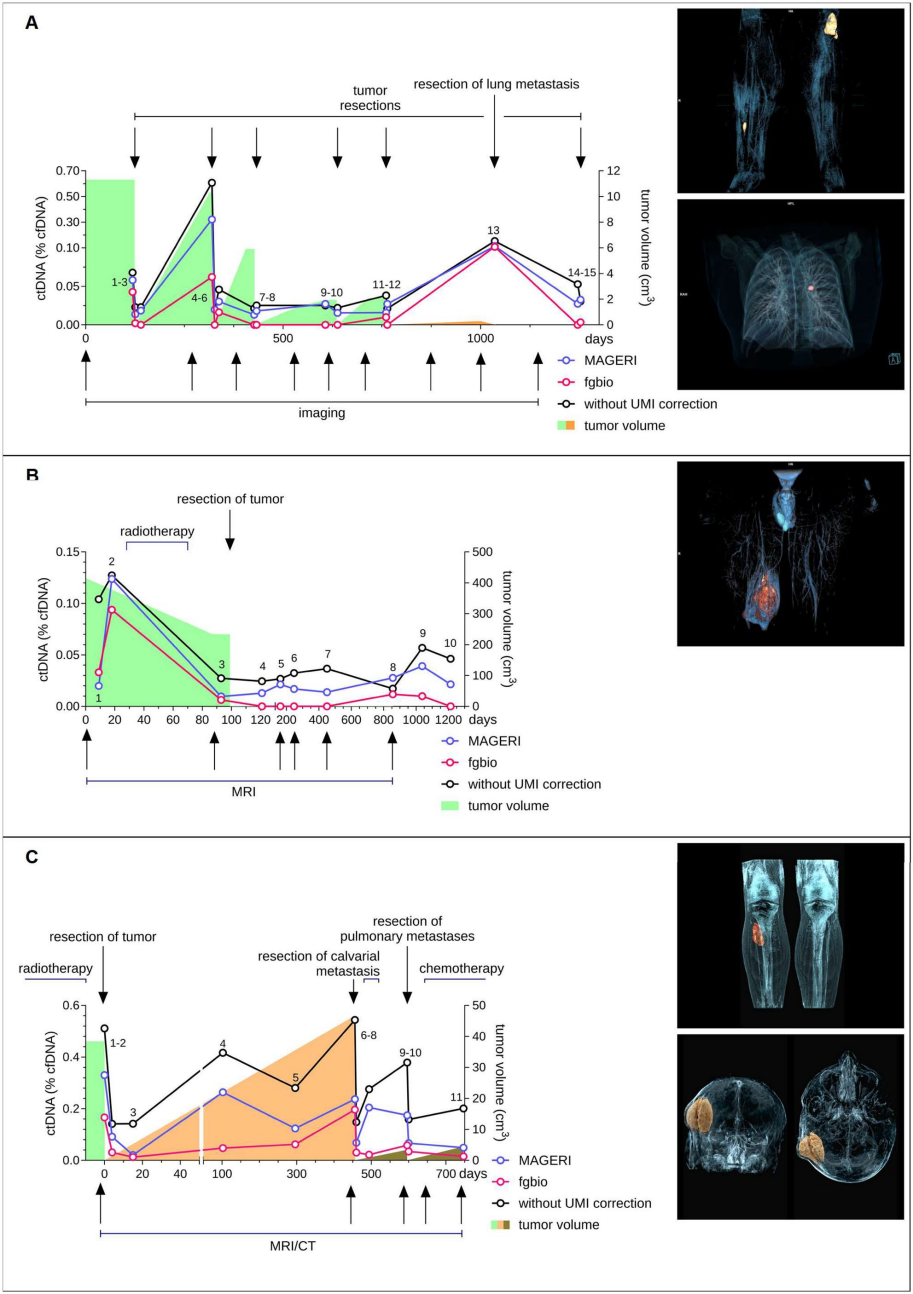
Comparison of different pipelines analyzing patients' plasma samples. To objectify the influence of the previously described differences, various pipelines were used to search for SNVs and SVs in patient plasma samples. For SVs, every read was considered, whereas for SNVs, only mutations with at least 2 variant reads were included. (A) Patient 1 was initially presented with two small recurrences of an MLS that had undergone multiple surgeries. The recurrences were completely resected in the first operation, but further recurrences occurred and were treated surgically again. Plasma samples were collected at 15 different time points during the entire observation period. The ctDNA detected is shown as a fraction of the total cfDNA. The analysis without UMI correction showed positive reads at every time point, whereas the analysis with *fgbio* was negative for ctDNA at several time points. Tumor volume is highlighted in green, except for a lung metastasis (orange). Patient 2 initially presented with a neoadjuvantly treated MLS of the right thigh. The tumor was completely resected, and the patient was followed up on a regular basis. During the observation period of this study, patient plasma samples were collected at 10 different time points and sequenced after patient‐specific target enrichment. Neither *MAGERI* nor *fgbio* showed any breakpoints. Analysis of the samples without UMI correction showed high ctDNA levels at each time point, even after surgery. The analysis with *fgbio* showed a good correlation of ctDNA levels with the clinical course at the preoperative stage. After surgery, ctDNA remained negative except for two time points. Follow‐up has shown no evidence of recurrence. In this case, too, the ctDNA results were very heterogeneous depending on the analysis method used. This figure demonstrates easy assay transferability to other translocation‐driven sarcomas as patient 3 initially presented with a synovial sarcoma (SS) in his right lower leg (green tumor volume). Over one year after complete resection, a calvarial metastasis was detected and subsequently resected (orange tumor volume). Several pulmonary metastases occurred in the further course and were resected each (brown tumor volumes). During the observation period of this study, patient plasma samples were collected at 11 different time points and sequenced after patient‐specific target enrichment. CtDNA was detected at each time point with any of the analysis pipelines and with ctDNA concentrations between 0.14% and 0.54% (without UMI correction). The use of UMIs and the differentiated analysis of SNVs and SVs, respectively, substantially increased assay sensitivity with *fgbio* returning ctDNA concentrations between 0.19% and 0.012%.

Patient 1 initially presented with two small recurrences of an MLS that had been operated on several times. The recurrences were completely resected in the first operation, but more recurrences occurred in the further course, each treated surgically again. Additionally, a pulmonary metastasis appeared, which again was surgically resected. Within the entire observation period of 3.5 years, plasma samples were collected at 15 different time points (Figure 4A). The differences in detected ctDNA result from different pipelines used for analyzing SNVs and SVs. After the first two resections, a substantial decrease in ctDNA concentration postoperation from 0.0428% to 0.0022% and from 0.0622% to 0% (samples 1, 2, 4, and 5) was observed with *fgbio*. The following two recurrences (samples 7–10) were not detected with *fgbio*. The resection of the next recurrence again showed a substantial decrease in ctDNA from 0.01% to 0% (samples 11–12). Strikingly, the small lung metastasis of less than 1 cm^3^ (sample 13) led to a marked increase in ctDNA compared to the localized leg tumors.

In addition, the differences in the concentration of detected ctDNA between the analytical methods used were remarkable, especially between different genomic regions. As an example, the analysis of *SOGA1* on chromosome 20 with *fgbio* showed no positive events over the entire observation period. In contrast, there were positive events with high numbers in nearly every sample when analyzing without UMI correction or with *MAGERI* (Figure S1D). Concerning all mutations, the analysis without UMI correction showed positive reads at each time point, whereas the analysis with *fgbio* was negative for ctDNA at several time points.

The second patient was initially presented with an MLS in his right thigh with a tumor volume of 414 cm^3^ (Figure 4B). The MLS was treated with neoadjuvant radiotherapy (samples 2–3) with a decrease of tumor volume to 233 cm^3^. The tumor was subsequently resected completely and showed a good pathologic response to radiotherapy with a tumor necrosis rate of over 90%. During the following 3.5‐year observation period, patient plasma samples were collected at 10 different time points (Figure 4B). The routine follow‐ups showed no signs of recurrence so far. CtDNA in plasma samples was quantified with the previously published patient‐specific *combined panel* [18]. With each pipeline, an increase in ctDNA concentration was observed prior to radiation therapy (samples 1–2). After radiotherapy, each pipeline returned a substantial decrease in ctDNA concentration with still measurable ctDNA. After complete resection (samples 4–10), ctDNA remained positive in every sample when analyzed with *MAGERI* or without UMI correction. Only *fgbio* remained negative for ctDNA after surgery (samples 4–7, sample 10) except for two samples (samples 8–9 with 0.011 and 0.009%, respectively). The analysis neither with *MAGERI* nor with *fgbio* revealed any breakpoints. Additionally, assay sensitivity was substantially increased with the use of UMIs. Without UMI correction, ctDNA was detectable down to 0.017%, while the analysis with fgbio still detected ctDNA at 0.006% (Figure 4B). For this observation period, *fgbio* showed the best correlation of ctDNA values to the clinical course. Further clinical follow‐up has so far shown no evidence of recurrence.

### 3.5 Transferring the Assay for Detection of ctDNA in Plasma Samples of Synovial Sarcomas

To see how the assay performs on other translocation‐driven sarcomas, the workflows and analysis pipelines were used to quantify SNVs and SVs in plasma samples of a patient with a synovial sarcoma (SS, patient 3). Every sample was analyzed with the *MAGERI* pipeline, *fgbio*, and without UMI correction. In the analysis of SVs, every read was considered, whereas for SNVs only mutations with at least 2 variant reads were included.

Prior to this study, WES was performed on tumor tissue and matched leukocyte DNA of patient 3 to identify patient‐specific variants [27]. These were combined with the patient‐specific translocation t(X;18) that was identified in a prior publication [27] to design another patient individually *combined panel* (Supporting Information). This allowed for targeting 18 genomic variations in cfDNA.

Patient 3 initially presented with a SS in his right lower leg, measuring 38 cm^3^ (Figure 4C). Over a 3‐year period, he attended our department for treatment and routine follow‐ups, during which we collected 11 plasma samples to quantify ctDNA. The patient underwent neoadjuvant radiotherapy, followed by complete tumor resection (Figure 4C, day 0). After resection, ctDNA levels dropped from 0.51% to 0.14% (samples 1–3, without UMI correction). Within months after the initial resection, an increase in ctDNA was noted despite the absence of tumor recurrence on CT and MRI scans (samples 4 and 5). Approximately 1 year later, a 44 cm^3^ calvarial metastasis was discovered and subsequently removed, followed by adjuvant radiotherapy. CtDNA levels showed elevated concentrations preresection and a substantial decrease postresection (samples 6 and 7). CtDNA concentration rose again with the detection of bilateral lung metastases and then dropped after incomplete resection (samples 8 and 9). Further lung metastases were resected, followed by adjuvant chemotherapy (samples 10 and 11).

The analysis with *MAGERI* showed extraordinarily high VAFs of up to 12% for reads on *ACOT11* and the associated high ctDNA concentrations of over 2.24% (Figure S3). The analysis neither with UMI correction nor with *fgbio* returned similar results with maximum VAFs on *ACOT11* of 0.12% and 0.092%, respectively, suggesting erroneous UMI handling of *MAGERI* for this region. For better comparability, *ACOT11* was, therefore, excluded from the final analysis (Figure 4C).

Comparing the different pipelines, none of the samples were negative for ctDNA. The use of UMIs led to a substantial improvement in sensitivity, with ctDNA concentrations ranging from 0.14% to 0.54% without UMI correction, whereas *fgbio* detected ctDNA with concentrations from 0.19% to 0.012%. *Fgbio* was the only pipeline showing a decrease in ctDNA concentrations from sample 7 (0.03%) to sample 8 (0.022%, adjuvant radiotherapy after calvarial metastasis) and from sample 10 (0.058%) to sample 11 (0.034%, adjuvant chemotherapy). Breakpoints were detected in sample 6 only. Five reads were detected without UMI correction, 3 reads with *fgbio*, and 2 reads with *MAGERI*. In conclusion, these results underline the advantages of the differentiated analytical approach with good transferability to other translocation‐driven sarcomas.

## 4 Discussion

Detection of local recurrence and distant metastasis is difficult in patients with MLS, as these tumors often have a distinct pattern of nonpulmonary spread throughout the body. Follow‐up examinations, thus, need to include extensive imaging, such as whole‐body MRI or CT scans [5]. Circulating tumor DNA is a potential biomarker of recurrence in patients after tumor resection and allows monitoring of tumor activity and treatment response in metastatic patients. As ctDNA has a short half‐life (approximately 2 h) it can allow an accurate snapshot of the genomic landscape of the tumor [28]. Previously, we quantified *FUS‐DDIT3* and the *TERT* promoter mutation C228T using real‐time PCR and droplet digital PCR (ddPCR) and showed a correlation between ctDNA concentrations, the clinical course, and tumor burden [17]. Despite these promising results, routine diagnostics were severely limited, as these techniques required fresh frozen tumor tissue, establishing patient individual qPCR assays, and could only detect a maximum of two mutations simultaneously. In a subsequent study, NGS with subtype and patient‐specific panels was employed. With these, mutational profiling could be achieved with small amounts of FFPE tumor‐DNA, and they enabled simultaneous quantification of multiple tumor mutations in cfDNA [18].

MLS is characterized by specific genetic translocations t (12;16) (q13;p16) and t (12;22) (q13;22q11‐12) which lead to the fusion of the DDIT3 gene to FUS or EWS [8, 9]. Despite these specific translocations, only a few additional hotspot mutations have been identified in MLS (*TERT* promoter, *PIK3CA*, *PTEN*, *TET2* and *TP53*) [10, 11, 12]. As demonstrated in a previous publication, targeting only hotspot regions with the subtype‐specific panel is not adequate for liquid biopsy as a diagnostic [18]. With an average of 2.8 somatic mutations per patient as possible targets, this assay showed insufficient specificity [18]. Therefore, we included individual SNVs detected via WES (patient‐specific panels). These additional targets led to significantly increased sensitivity, highlighting the importance of SNVs for better test performance of liquid biopsy in MLS. As a major disadvantage, ultra‐deep sequencing leads to an artifact rate of approximately 0.1% [29]. Since the concentration of cfDNA in plasma samples is also well below 0.05% of the total cfDNA (Figure 4), the comparatively high artifact rate has a negative impact on the specificity of the test performance in terms of false‐positive events (Figures 1, 2, 3).

In this study, we performed an in‐depth bioinformatic analysis to improve the test performance. This standardized pipeline will allow monitoring of disease activity in translocation‐associated sarcomas in a routine diagnostic setting. The provided diagnostic assay and analysis pipeline demonstrated markedly improved specificity without compromising its sensitivity. For the analysis of ctDNA, many different approaches have been investigated with regard to the use of UMIs [6]. Different sequencing parameters have influenced the outcome of the analysis and, with that, the display of the clinical course when analyzing plasma samples from patients. In particular, the processing of the UMI adaptors and the way in which the consensus reads are generated have an influence on the coverage and depend crucially on it. This became evident when we used different analysis pipelines with different settings, with *fgbio* delivering the best results overall (Figures 1, 2, 3 and S3B). False‐positive events were not observed in this study regarding patient‐specific translocations in MLS. For this reason, when analyzing SVs with UMIs, a less stringent selection could be made to filter out artifacts to obtain higher coverage and still achieve a high degree of specificity.

Another very effective measure to improve specificity was to consider only mutations with at least 2 variant reads for SNVs. This has significantly reduced the rate of false‐positive events in various pipelines. But here, too, the analysis of SVs could be carried out differently. Due to the low probability of false‐positive events in SVs, each variant read was considered to obtain higher sensitivity with still a high degree of specificity. Whether an improvement in the analysis of SNVs using duplex consensus calling is possible will be investigated in the future [19]. Nevertheless, the differentiated approach in the analysis of SNVs and SVs in this study enabled reliable detection of ctDNA that, in turn, showed a good correlation to the clinical course of the patients' disease.

Each MLS tumor harbors a unique breakpoint sequence within introns of *DDIT3*, *FUS*, and *EWS*, forming the t(12;16) and t(12;22). Due to the position in introns, the breakpoints are not covered by WES, which we use for mutational profiling of SNVs. We, therefore, employed a subtype‐specific panel to cover the breakpoint regions to sequence and validate each individual breakpoint sequence. In addition to SNVs, this allows tracking of highly tumor‐specific breakpoints and further increases the sensitivity of the assay. The approach of using subtype‐specific target enrichment to simultaneously detect different translocations with a single panel can easily be transferred to any translocation‐driven tumor. To increase the sensitivity by simultaneously tracking multiple tumor mutations, we additionally included genes in the panel that are commonly altered in MLS, such as *PIK3CA* and the *TERT* promoter region [18]. As an example of another translocation‐driven sarcoma, the assay performance was also demonstrated on plasma samples of synovial sarcomas (Figure 4C) [27]. The possibility of sequencing both breakpoints and point mutations simultaneously with one panel allows a differentiated approach in further analysis to obtain the best possible sensitivity and specificity.

Tumor mutations were identified by comparing the tumor exome to the matched leukocyte control sample. Mutations that are described as pathogenic, for example, in COSMIC (https://cancer.sanger.ac.uk/cosmic), were included in the individual *combined panels* together with the mutations identified in the *standard panel*. We technically adapted and optimized the technique by, firstly, enriching ctDNA; secondly, decreasing false positives using molecular barcodes [30]; and thirdly, increasing the on‐target rate by double hybridization capture [19]. By employing these techniques, NGS allowed the detection of mutations with a VAF of less than 0.05%.

A major disadvantage of our approach is the need for matched tumor tissue. Especially in the case of external tumor resections, assuming a benign tumor, the so‐called “whoops procedure,” there is often not enough tumor tissue available after subsequent wide resection for a mutation analysis. An approach that does not require corresponding tumor tissue would have the great advantage that this patient group could also be included and the costs for mutation analysis could be saved. Improved bioinformatic breakpoint detection could enable the detection of breakpoints directly from patient plasma in the future due to its high specificity. For SNVs, this appears to be much more difficult due to other causes, such as clonal hematopoiesis. Another disadvantage is the patient‐specific approach. This enables a high level of specificity and safety in the event of sample mix‐ups. On the other hand, despite largely automated mutation analysis and panel creation, a considerable amount of human labor is still required. In the future, a combined approach of WGS sequencing of plasma cfDNA in combination with deep learning and usage of artificial intelligence may offer a way to partially eliminate these disadvantages [31].

## Author Contributions

**A. Schmid:** conceptualization (equal), data curation (equal), formal analysis (equal), methodology (equal), software (equal), visualization (equal), writing – original draft (equal). **U. Lausch:** data curation (equal), methodology (equal), writing – review and editing (equal). **A. Runkel:** data curation (equal), formal analysis (equal), methodology (equal), writing – review and editing (equal). **J. Kiefer:** data curation (equal), formal analysis (equal), writing – review and editing (equal). **T. Pauli:** methodology (equal), validation (equal), writing – review and editing (equal). **M. Boerries:** methodology (equal), validation (equal), writing – review and editing (equal). **B. Bogner:** data curation (equal), visualization (equal), writing – review and editing (equal). **S. U. Eisenhardt:** conceptualization (equal), data curation (equal), formal analysis (equal), supervision (equal), writing – original draft (equal). **D. Braig:** conceptualization (equal), data curation (equal), formal analysis (equal), project administration (equal), supervision (equal), writing – original draft (equal).

## Ethics Statement

The Ethics Committee of the Albert‐Ludwigs‐University of Freiburg, Germany, approved the study (study number: 236/16). The design and performance of the study are in accordance with the Declaration of Helsinki. Participants signed informed consent before inclusion, allowing analysis of tumor tissue, blood samples, and clinical data.

## Conflicts of Interest

The authors report no Conflicts of Interest.

## Supporting information


Data S1.

## Data Availability

Sequencing data can be made available upon reasonable request.
