# Supplementary material for: Improved Quantification of Circulating Tumor DNA in Translocation‐Associated Myxoid Liposarcoma by Simultaneous Detection of Breakpoints and Single Nucleotide Variants
Source: Cancer Med. 2025 Feb 20;14(4):e70704. doi: 10.1002/cam4.70704 (PMC11842865; doi:10.1002/cam4.70704)
Supplement: Supplementary file 1 — Data S1. [file CAM4-14-e70704-s001.pdf]

Supplementary figures

Supplementary figure 1

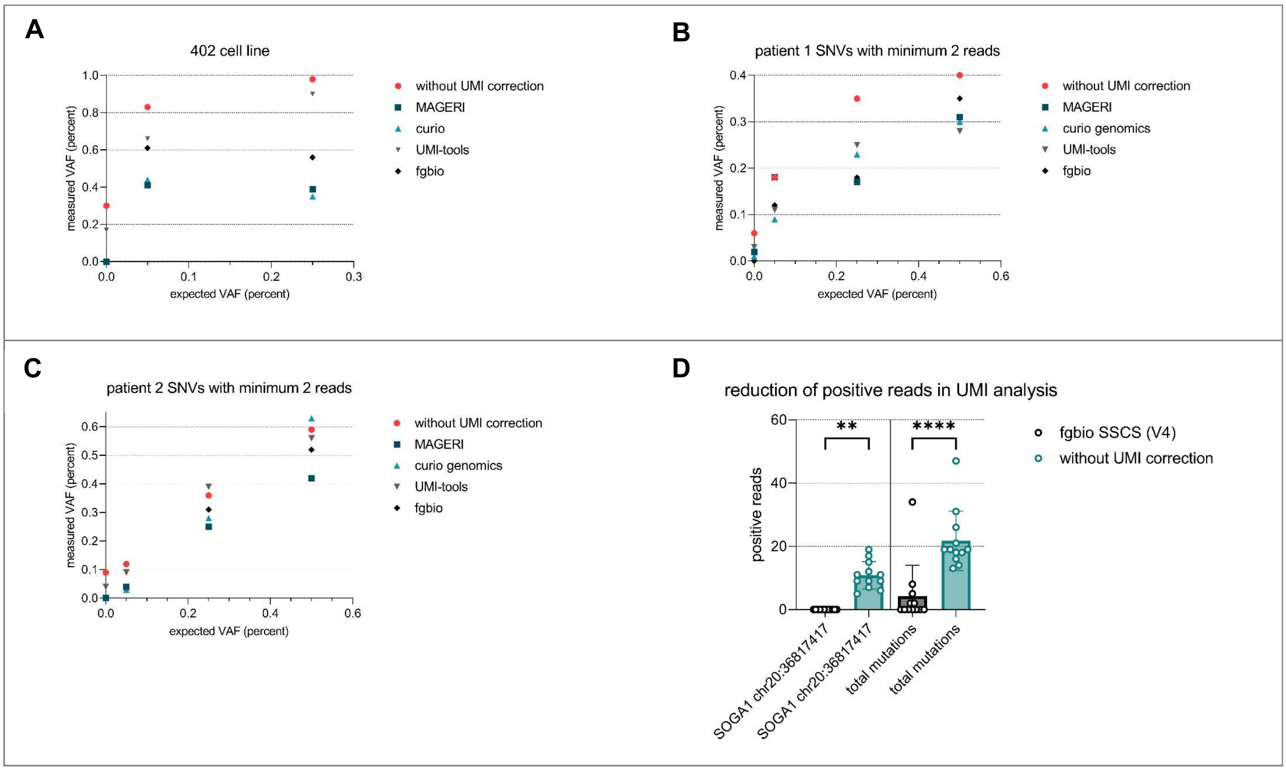

(A) Shown is the measured VAF compared to the expected VAF in percent. Only SNVs are considered for this figure. Only variations with at least two mutated reads were counted. Due to the different scale, the samples with 2.5% VAF were excluded for better visualization. **(B-C)** These figures also show the measured VAF compared to the expected VAF in percent for patient 1 and 2. The fgbio pipeline shows the best correlation between expected and measured VAF. **(D)** Supplementary data to Figure 4 (A). The difference in this case is largely due to a point mutation in SOGA1. In the analysis without UMI correction, this mutation was detected at a comparatively high level at every time point. At the same time, the analysis with fgbio filtered out this mutation completely, leading to a significant reduction of positive reads.

Supplementary figure 2

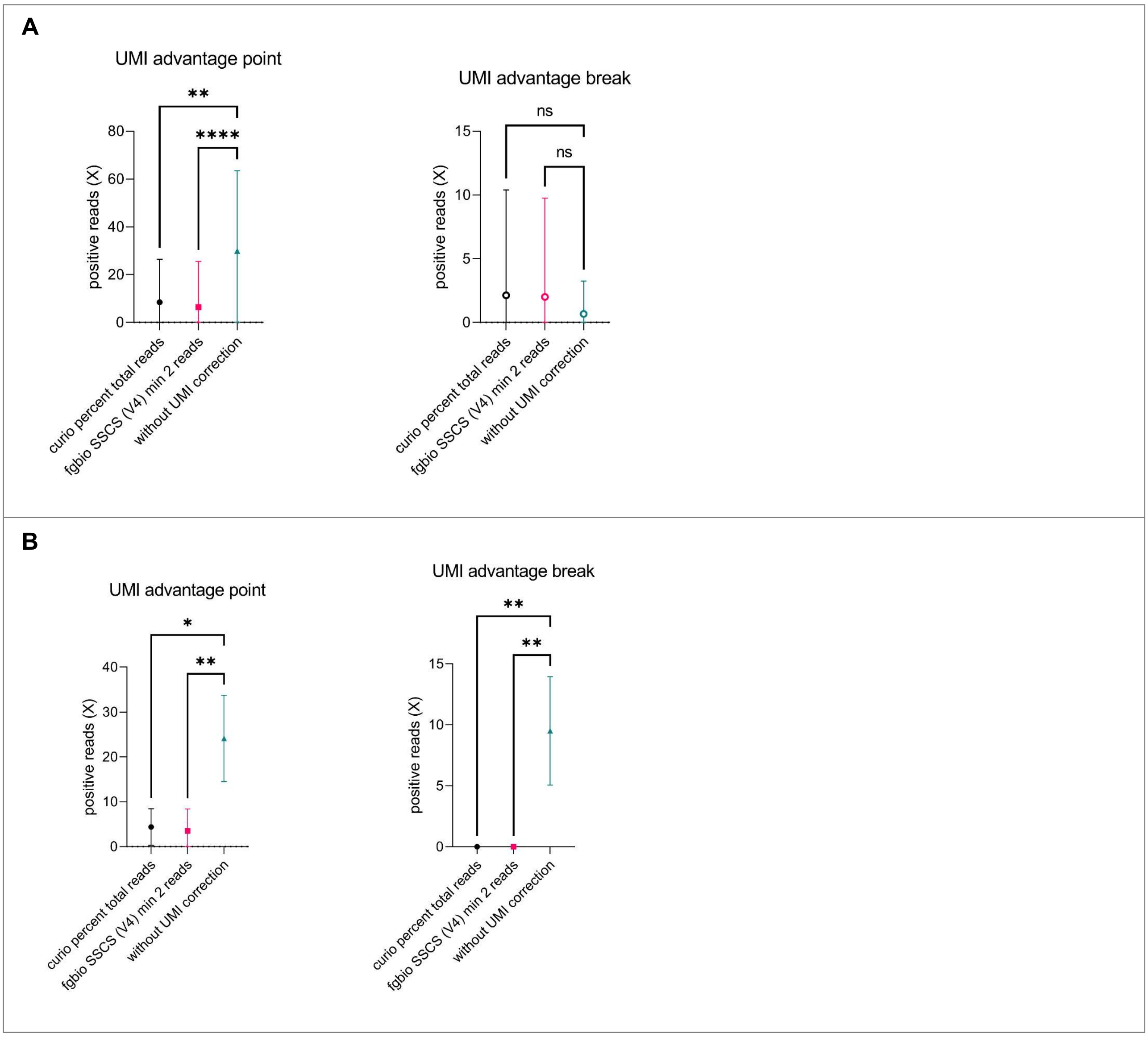

The differences in the number of positive reads for the different analysis methods in patient plasma samples are shown separately for each time point. SNVs and SVs were analyzed separately (see Figure 4). **(A)** Shows the difference for the different plasma samples of patient 1. It is clear from the comparison of SNVs to SVs that the use of UMIs does not result in a significant reduction in (false) positives for SVs. **(B)** Shows the differences for the different plasma samples from patient 2. When analyzing the SVs without UMI correction, the disproportionately high coverage with an equally high number of positive reads was striking.

Supplementary figure 3

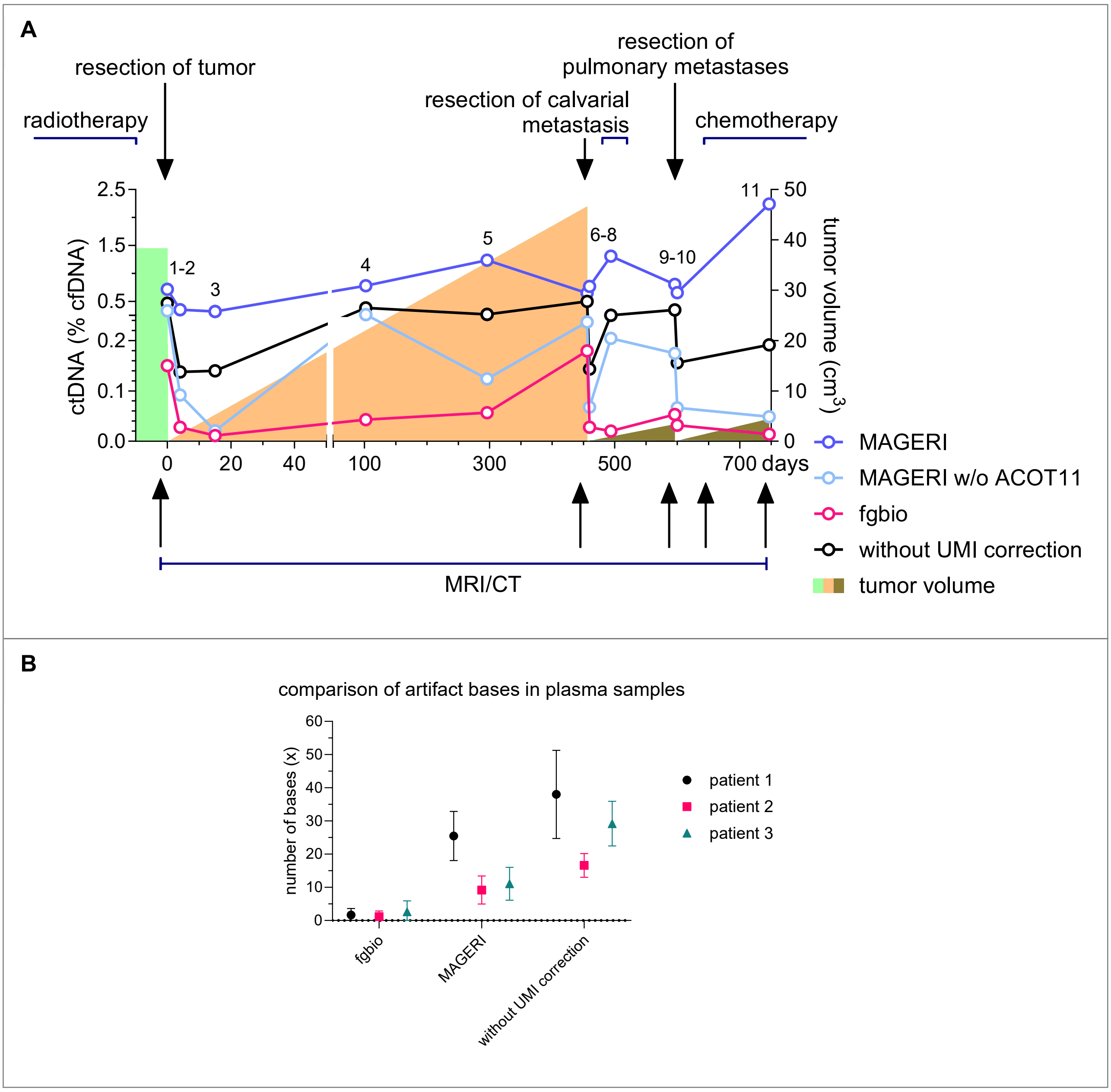

(**A**) 11 plasma samples from patient 3 with a Synovial Sarcoma (SS) were analyzed for ctDNA (**Figure 4 C**). Targets were enriched using a patient specific *combined panel*. SVs and SNVs were detected separately with different analytical pipelines (*MAGERI*, *fgbio* and without UMI correction). CtDNA was detected at each time point with any of the pipelines. The analysis with *MAGERI* showed extraordinarily high VAFs of up to 12 % for reads on *ACOT11*, resulting in ctDNA concentrations of up to 2.24 %. The analysis neither with *fgbio* nor without UMI correction showed similar results for reads on *ACOT11*. For this reason, the analysis with *MAGERI* was also carried out again with the exclusion of *ACOT11 (MAGERI w/o ACOT11)*. **(B)** The positive benefit of UMIs can also be described by the number of artifact bases. For this purpose, in all plasma samples of patients 1-3, the bases that correspond

neither to the reference nor to the variant were counted for each position. Again, the different analytical pipelines were compared to each other with *fgbio* resulting in the greatest reduction in artifacts.
